# Crashworthiness Prediction of Perforated Foam-Filled CFRP Rectangular Tubes Crash Box Using Machine Learning

**DOI:** 10.3390/polym17212887

**Published:** 2025-10-29

**Authors:** Harri Junaedi, Khaled Akkad, Tabrej Khan, Marwa A. Abd El-baky, Mahmoud M. Awd Allah, Tamer A. Sebaey

**Affiliations:** 1 Department of Engineering Management, College of Engineering, Prince Sultan University, Riyadh 12435, Saudi Arabia; tkhan@psu.edu.sa (T.K.); tsebaey@psu.edu.sa (T.A.S.); 2 Department of Industrial Engineering, Faculty of Engineering, King Abdulaziz University, Jeddah 21589, Saudi Arabia; kakad@kau.edu.sa; 3 Industrial Engineering Department, College of Engineering, King Khalid University, Abha 61421, Saudi Arabia; msallam@kku.edu.sa; 4 Center for Engineering and Technology Innovations, King Khalid University, Abha 61421, Saudi Arabia; 5 Department of Mechanical Design and Production Engineering, Faculty of Engineering, Zagazig University, Zagazig 44519, Egypt; mmostafa@eng.zu.edu.eg; 6 Mechanical Engineering Department, Southern Methodist University, Dallas, TX 75240, USA

**Keywords:** hybrid structures, thin-walled structure, crushing test, energy absorption, regression algorithm

## Abstract

The use of carbon fiber-reinforced polymer (CFRP) tubes as crash boxes has become a subject of interest due to their high specific strength and energy absorption capabilities. This study investigates the crashworthiness performance of rectangular tubes made of CFRP, with and without holes and polyurethane foam (PUF)-filled inner structures. The designed tubes were subjected to quasi-static axial compression loading. In addition to carefully documenting failure histories, data on crash load and displacement responses were methodically recorded during testing. To evaluate crashworthiness performance, three design parameters were considered: hole diameter, the number of holes in both the x and y directions, and whether the tube was filled with foam or left unfilled. Machine learning (ML) was also used to reduce the time and cost by predicting the crashworthiness indicators of the tubes from fewer experiments. A collection of ML algorithms such as decision tree regressor (DTR), linear regressor (LR), ridge regressor (RR), lasso regressor (LAR), elastic nets (ENs), and multi-layer perceptron (MLP) have been utilized to predict crashworthiness indicators such as initial peak force (*P_ip_*), mean crushing force (*P_m_*) and energy absorption (*EA*) of the design tubes from the experimental data. The experimental results showed that PUF-filling significantly enhanced crashworthiness properties, with *P_m_* and *EA* increasing by nearly threefold compared to unfilled tubes. Furthermore, in unfilled tubes, the introduction of holes led to varying effects depending on the hole diameter and placement. Meanwhile, in PUF-filled tubes, the presence of holes reduced the crashworthiness performance. For ML prediction, the DTR achieved the best accuracy with the lowest value of root mean squared error (RMSE) and mean absolute percentage error (MAPE) of 1251 and 11.37%, respectively. These findings demonstrate both the importance of PUF-filled, perforation configurations and the feasibility of ML models in optimizing CFRP crash box designs.

## 1. Introduction

In vehicle crashworthiness design, choosing the right materials and structural shapes for the crash box is essential for efficiently managing impact energy during a collision [1,2]. The main objective is to facilitate controlled deformation of the vehicle, allowing it to dissipate crash energy while reducing the forces transferred to the occupants [3,4]. Material selection requires balancing multiple factors, including energy absorption, weight reduction, and cost. Crash box structures have been made from many different materials, such as steel, aluminum, glass fiber-reinforced polymer (GFRP), Carbon fiber-reinforced polymer (CFRP), and various hybrid combinations of these materials [5]. Each material has benefits as well as drawbacks.

Metal alloys such as steel and aluminum alloys are widely preferred for their effective impact energy absorption. These materials undergo controlled deformation during a crash, dissipating energy and reducing the forces transmitted to vehicle occupants, thereby enhancing safety [6,7]. Steel tubes are still the most common type of crash box because they are strong, flexible, and can absorb energy steadily as they bend. However, their higher density leads to heavier vehicles and reduces fuel efficiency. Aluminum alloy tubes are another option for lightweight, crashworthy components due to their significantly lower weight compared to steel. Aluminum hollows absorb substantial energy per unit mass; however, they do not function as effectively as advanced fiber-reinforced composites in terms of specific energy absorption [8].

In recent years, polymer-based composites have gained prominence in industries such as automotive and aerospace due to their exceptional combination of specific strength, stiffness, and energy absorption capabilities [9,10,11]. Polymer-based composites offer significant advantages in energy-absorbing applications, where reducing weight while maintaining structural integrity is essential [12,13,14,15]. Being considerably lighter than metals, they contribute to improved fuel efficiency and overall vehicle performance. Additionally, their energy absorption properties enable effective dissipation of impact forces during a collision, playing a vital role in vehicle safety.

Researchers have looked into using GFRP tubes as energy-absorbing parts since they are relatively low-cost and easy to fabricate. GFRPs possess a lower density than metals; nonetheless, their mechanical properties and crashworthiness are typically inferior to those of CFRP [16]. CFRP, on the other hand, is a widely used polymeric composite material in high-performance applications, such as automotive and aerospace, due to its exceptional strength-to-weight ratio and durability [17,18]. In such applications, the structural integrity of CFRP components is critical, especially in scenarios involving crash or impact. CFRP tubes have better specific energy absorbed (*SEA*), higher specific stiffness, and better tailorability owing to fiber orientation and laminate design compared to GFRP and metals. Because of this, CFRP is an auspicious material for crash boxes, especially in race cars and planes, where weight is essential and energy absorption needs to be maximized while keeping weight to a minimum. Nevertheless, the comparatively elevated cost of carbon fibers compared to steel, aluminum, and GFRP composites constrains the extensive utilization of CFRP to niche applications.

However, introducing holes in CFRP crash box structures is often unavoidable, whether due to design requirements or manufacturing constraints. These holes can significantly influence the crashworthiness of the material, potentially reducing its ability to absorb energy during an impact. One promising approach that has been reported to enhance the crashworthiness of the CFRP is using foam-filled tubes [19,20]. Adding foam to the inner part of the tube can improve energy absorption and redistribute stresses around the holes, thereby compensating for the structural weaknesses caused by the discontinuities. Studying the combined effects of holes and foam-filled reinforcement on the crashworthiness indicators of CFRP structures is important. Understanding these interactions can provide valuable insights for optimizing the design of CFRP components to ensure both functionality and safety in high-performance applications.

Additionally, the integration of machine learning (ML) has emerged as a transformative tool across various scientific and technological domains. The ability of this tool to predict different types of data has become more attractive in recent years. ML has become a powerful tool in materials science and engineering for predicting the mechanical properties of materials. Experimental work in the materials science and engineering field is time-consuming, resource-demanding, and costly. The ML approach can provide a faster way of analyzing the patterns and correlations between material compositions, processing parameters, and mechanical properties [21]. Different ML approaches, such as decision tree regression, neural networks, and support vector regression, are commonly employed to handle complex, non-linear relationships characteristic of material behaviors [22]. It can accelerate the process by rapidly predicting mechanical properties and narrowing the focus to the most promising candidates for further study.

ML has revolutionized the analysis and optimization of polymer composite laminates, particularly in predicting mechanical behaviors and enhancing design efficiency. The integration of ML techniques allows for significant advancements in understanding the properties and performance of these materials. Kazi et al. [23] used an Artificial Neural Network (ANN) to optimize the effect of cross-section aspect ratio on the crashworthiness of cotton fiber-epoxy composite. The prediction obtained from the ANN was consistent with the experimental results. Borse et al. [24] utilized a reinforcement learning (RL)-based optimization technique to design a crash box. This structural model is explored in terms of the shell stability phenomenon under dynamic axial loading circumstances. The numerical approximation model is constructed and simulated using Finite Element simulation. Therefore, it was suggested that the RL agents be trained for a longer time to reduce this fluctuation. Regardless, the framework can predict the optimal thickness. Altogether, the framework can be normally used to optimize the thickness of a square crash box of defined length and cross-section and for specific crash tests and crashworthiness metrics. Liang et al. [25] investigated a machine learning-based multi-objective optimization of pinecone-inspired multi-celled tube crashworthiness indicators based on existing experiments. Machine learning algorithms were used to design alternative models of these tubes. A non-dominated sorting genetic algorithm II (NSGA-II) approach was employed to perform a multi-objective optimization. Moreover, the specific absorbed energy (SEA) of the optimum design from the ML NSGA-II approach showed an average variation of 5.91% from the experiment result.

ML algorithms, such as ANN, have been effectively employed to predict the mechanical behavior of laminated fiber-reinforced polymer composites, achieving high accuracy and reduced computational costs [26]. While ML offers significant advancements in predicting mechanical properties, challenges remain in model selection and data availability, necessitating further research to fully harness its potential in composite material design. Multilayer Perceptron (MLP) and Recurrent Neural Networks (RNN) have been developed to estimate stiffness and fatigue life, demonstrating excellent predictive capabilities for polymer matrix composite laminates by Arnold et al. [27]. Data-driven ML models have been utilized to assess the fatigue response of carbon fiber-reinforced polymer composite laminates, capturing non-linear load–displacement relationships with minimal error [28]. Additionally, ML approaches have been proposed to predict damage behavior under out-of-plane loading, offering a cost-effective alternative to traditional experimental methods [29].

The literature review in this study reveals a significant gap in experimental research concerning the impact of perforations and foam-filled reinforcements on the crashworthiness performance of composite material structures and their predictive modeling using Machine Learning (ML). To address this gap, the present work aims to experimentally evaluate the crashworthiness of perforated rectangular CFRP tubes, both with and without PUF-filled inner structures. Additionally, different ML techniques were trained from the experimental data and used to predict crashworthiness indicators such as initial peak force, mean crushing force, and energy absorption, given the inputs of circular hole sizes, hole arrays, and PUF-filled. This is important for cost reduction as ML uses different values of the previously mentioned input. These advantages eliminate the need for further experimentation with varying holes, arrays, and PUF sizes. This study also explores the effectiveness of various ML techniques in predicting crashworthiness, validating their accuracy against the experimentally obtained data.

## 2. Materials and Methods

### 2.1. Materials

The sample used for testing was a hollow tube with a rectangular cross-section, measuring 52 mm × 27 mm on the outer dimensions. The tube had a wall thickness of 1.15 mm and a length of 50 mm, forming a structure that combines high-strength materials with lightweight characteristics. This hollow tube is made from CFRP, which is known for its excellent strength-to-weight ratio, durability, and corrosion resistance. The tube’s construction features a braided CF layer on the outer surface and unidirectional CF arranged inside (Toray T300, Toray Composite Materials America, Inc., Tacoma, WA, USA). The orientation of these fibers follows a specific stacking sequence of [±45/0]_s_, which refers to a quasi-isotropic layup where layers are oriented at ±45° to the longitudinal axis and at 0° for the central ply. This specific fiber arrangement is chosen to provide balanced mechanical properties in both the longitudinal and transverse directions, improving the tube’s ability to resist various loading conditions. The fiber volume fraction (V_f_) of the laminate is approximately 50%, indicating that half of the volume is made up of fibers, which enhances the structural properties and stiffness of the composite while keeping the tube relatively lightweight. The CF has tensile strength and modulus of 3500 MPa and 230 GPa, respectively. The matrix material is a thermosetting epoxy resin 304 (Mitsubishi Chemical Carbon Fiber and Composites, Sacramento, CA, USA), which provides adhesion between the fibers. The epoxy resin used possesses a glass transition temperature (Tg) of 140 °C, a tensile strength of 66 MPa, and an elastic modulus of 3.7 GPa. Filling the interior of the CFRP tube is a two-component rigid PUF from Totalboat (Bristol, RI, USA). The foam is used as an inner filler to enhance the tube’s structural integrity by providing additional internal support and helping to distribute internal stresses more uniformly. The foam also contributes to reducing the overall weight of the tube while improving the energy absorption capacity of the structure. The cured density of the PUF is 90 kg/m^3^, making it an extremely light material, further reducing the overall weight of the composite tube. The foam’s low density also adds to the overall energy dissipation properties of the tube, which may be critical for specific applications.

### 2.2. Experimental Methods

**Figure 1:**
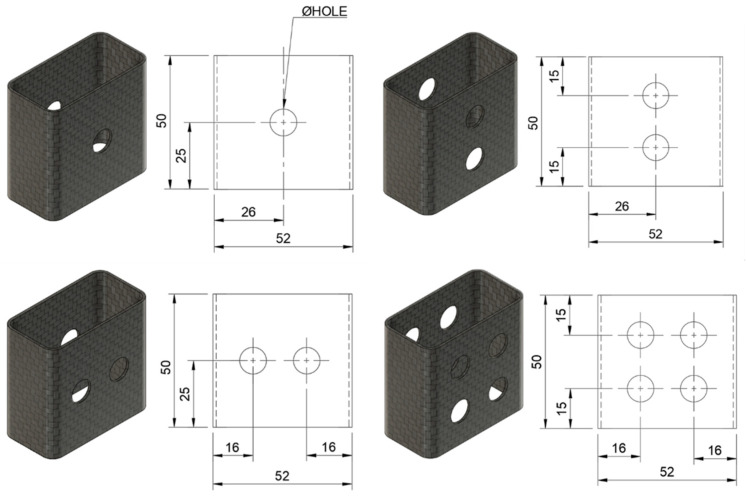
Sample configurations and dimensions.

The effect of holes on the crashworthiness indicators of tubes with four different parameters was studied, e.g., hole diameter (4, 6, 8, and 10 mm), PUF-filling (with and without PUF), number of holes in the x-direction (1 and 2 holes) and number of holes in the y-direction (1 and 2 holes), resulting in a total of 32 sample configurations. The four diameter levels were selected to represent a practical range relevant to real-world applications, from a minimal size to the maximum feasible without causing premature failure. The number of holes was constrained by the specimen’s geometry to ensure structural integrity during testing; accommodating more than two holes was not feasible. From the total of 32 sample configurations, fourteen selected distinct sample configurations were experimented, each with three repetitions, leading to a total of 42 data points. The configurations and dimensions of the samples are detailed in Figure 1 and Table 1, which provide a clear overview of the different tube configurations used in this study. The configurations likely differ in parameters such as the orientation of the fibers, wall thickness, foam filling, and hole placement, all of which influence the crash performance of the tubes. The unfilled tubes were directly drilled. The drilling was carried out using a mechanical drill to create holes according to the specific configuration described. This drilling procedure was carried out with precision to ensure consistency across the different configurations, as the drilled holes significantly impact the structural integrity and failure modes of the tubes during crash tests. The precise placement and size of these holes are essential for evaluating how the tube behaves under crash loading, as the holes can act as stress concentrators, influencing how the material deforms or absorbs energy [30]. To minimize drilling-induced delamination, a wood backing support was placed under the tube’s wall during the drilling process [31]. After drilling, the edges of the holes were carefully smoothed with sandpaper to remove any roughness or imperfections caused by the mechanical drilling process. This sanding step was essential to reduce any potential sharp edges or stress risers that could lead to premature failure during crash testing.

For the PUF-filled tubes, the preparation process was slightly different. The procedure began by sealing one end of the tube with adhesive tape, ensuring that the foam mixture would not escape during the filling process. After thoroughly mixing the two components of the rigid PUF, the liquid foam mixture was poured into the inner cavity of the tube. The tubes were then allowed to cure for 48 h at room temperature, ensuring that the foam fully solidified and adhered to the interior surface of the tube. This curing time was critical to ensuring that the foam would develop its final mechanical properties, including its low density and energy-absorbing capabilities. Once the PUF foam was fully cured, holes were drilled into the filled tubes. Drilling through both the foam and the CFRP laminate required careful attention, as excessive force during drilling could cause delamination of the CFRP material or disruption of the foam structure. The hole edges were sanded after drilling, following the same procedure as for the unfilled tubes.

### 2.3. Crashworthiness Indicators

**Figure 2:**
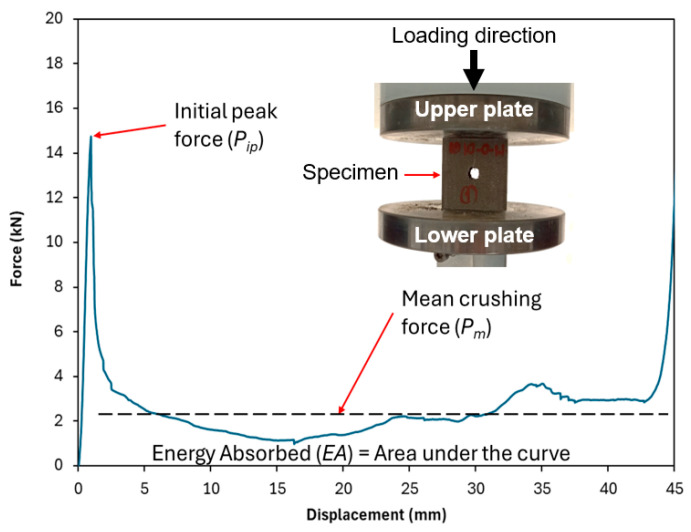
A sample for compressive force-displacement curve for the tested specimens.

The crashworthiness performance of the tubes was assessed through quasi-static compression testing. This test provides valuable comparative data, allowing for a relative assessment of different designs. Furthermore, the tests reveal fundamental properties, such as the basic crushing mechanism and the stability of progressive failure. However, a limitation remains, as the material’s performance under quasi-static compression testing may differ from its behavior during an actual impact collision, as the high strain rates can cause increased brittleness, leading to a high peak force or catastrophic shattering instead of controlled, progressive crushing. In accordance with established testing protocols from previous studies on the crashworthiness of similar tubular structures, the crosshead speed was set to 5 mm/min. This speed was specifically chosen to replicate the quasi-static loading conditions [11,32]. Each specimen was placed between two rigid, flat steel plates and subjected to axial compression until reaching a predefined maximum displacement of 45 mm. This setup ensured uniform loading and minimized boundary effects that could influence the structural response. Throughout the testing process, force-displacement data were continuously recorded using a high-precision load cell and displacement sensor, ensuring accurate measurement of mechanical response parameters. As demonstrated in Figure 2, a representative force-displacement curve was obtained, capturing key crashworthiness characteristics. This real-time data acquisition facilitated a comprehensive analysis of the tubes’ structural behavior, including their load-bearing capacity, peak force, energy absorption capability, and failure mechanisms.

The crashworthiness of the tubes is assessed through the force-displacement curve obtained from the crushing test. These indicators include total initial peak force (*P_ip_*), mean crushing force (*P_m_*), and energy absorption (*EA*). These factors characterize the material’s ability to absorb energy during impact forces. *P_ip_* corresponds to the first peak after the linear part of the force (*P*)-displacement (*δ*) curve. *P_m_* represents the average force along the displacement between the initial peak force and the maximum displacement. Energy Absorption (*EA*) is measured by calculating the area under the force-displacement curve, as shown in Equation (1).

(1) EA=∫Pdδ

Other properties, such as specific energy absorption (*SEA*) and crush force efficiency (*CFE*), were calculated from the above three parameters. *SEA* was computed by normalizing the energy absorption by the total mass of the tube (*m*), as shown in Equation (2). Meanwhile, *CFE* was determined by the ratio of the mean crushing force to the initial peak force, as shown in Equation (3).

(2) $$SEA=\frac{\int Pd\delta }{m}$$

(3) $$CFE=\frac{P_{m}}{P_{ip}}$$

### 2.4. Machine Learning

The methodology for implementing machine learning (ML) methods to predict crashworthiness indicators follows a structured approach. A dataset comprising 42 entries (corresponding to 14 experimental setups, each repeated three times) was used for training, as detailed in Table 1. Since the dataset was already well-structured, with no missing values or inconsistencies, no additional preprocessing was required. To develop predictive models, a diverse set of ML algorithms was employed, including the Decision Tree Regressor (DTR), Linear Regressor (LR), Ridge Regressor (RR), Lasso Regressor (LAR), Elastic Net (EN), and Multi-Layer Perceptron (MLP). These algorithms were chosen to explore different regression approaches, ranging from simple linear models to more complex non-linear and regularized techniques, ensuring robust predictions. All ML models were implemented and trained using Python 3, leveraging the Scikit-learn library for efficient model development, training, and evaluation [33,34].

Each of the ML techniques was trained using a bootstrapping approach, where 10 different samples were generated with replacement, utilizing NumPy [35] random seeds ranging from 0 to 9. This approach ensured variability in the training process while maintaining consistency in reproducibility. Each sample contained a unique combination of training and validation splits, with the dataset partitioned into 80% for training and 20% for validation. This ratio was selected to balance model learning while retaining sufficient data for evaluating generalization performance. To assess the predictive accuracy of the ML models, two key error metrics were employed: the root mean squared error (RMSE) and the mean absolute percentage error (MAPE), as defined in Equations (4) and (5), respectively. These metrics were computed for each algorithm across the 10 generated samples, and the average validation RMSE and MAPE values were used as primary indicators of model performance [36].

(4) $$RMSE=\sqrt{\frac{1}{n}\sum_{i=1}^{n}{\left(A_{i}-P_{i}\right)}^{2}}$$

(5) $$MAPE=\left(\frac{1}{n}\sum_{i=1}^{n}\frac{\left|A_{i}-P_{i}\right|}{A_{i}}\right)\cdot 100\%$$

where $A_{i}$ and $P_{i}$ are the actual and predicted values, respectively, and *n* is the total number of observations in the validation set.

RMSE was chosen as it provides a direct measure of the average magnitude of prediction errors, penalizing larger deviations more heavily. MAPE, on the other hand, expresses the error as a percentage of the actual values, making it useful for comparing prediction accuracy across different scales. By averaging these metrics across multiple training samples, the robustness and generalization capability of each ML technique could be effectively evaluated. Different combinations of hole diameter, number of holes, and PUF-filled were used as the independent variables for predicting crashworthiness indicators. The output-dependent variables were *P_ip_*, *P_m_*, and *EA*. Meanwhile, other properties, such as *SEA* and *CFE*, were not predicted since they are derived from the three aforementioned properties. The testing set contains input parameters with unknown output values. These output values were predicted using ML algorithms, which will be shown in the results section later. Table 2 contains the input values in the testing set.

It is important to note that Table 2 presents input parameter combinations that differ from those in the training set. This deliberate variation allows the ML models to generalize beyond the trained data, enabling the prediction of crashworthiness indicators for new, unexplored configurations. By leveraging ML predictions, the need for conducting additional physical experiments for these new combinations is minimized, thus reducing time, cost, and resource consumption. The best-performing ML technique based on the validation results was used to predict the outputs, *P_ip_*, *P_m_*, and *EA*. The accuracy and reliability of these predictions were further examined and validated in the results section, where a comparative analysis between predicted and actual experimental values was conducted.

#### 2.4.1. Decision Tree Regression

Decision Tree Regression (DTR) is a supervised machine learning algorithm that predicts target values by recursively partitioning the dataset into smaller subsets based on input feature values [37,38]. At each node, the algorithm selects the optimal feature and corresponding threshold that minimizes a chosen loss function, typically the MSE for regression tasks. This hierarchical splitting continues until predefined stopping criteria are met, such as a minimum number of samples per leaf node or a maximum tree depth. For a given new input, the decision tree assigns it to a leaf node based on the learned splits, and the predicted output is calculated as the average of the target values of all training samples within that leaf node. This approach allows decision trees to effectively model complex non-linear relationships between input features and target variables. Decision tree regression has been widely applied across various engineering and materials science domains, particularly in predicting material properties of composite materials and polymers. These models are advantageous due to their ability to handle non-linear dependencies, interpretability, and relatively low computational cost. In this study, decision tree regression was employed as one of the ML techniques to predict key crashworthiness indicators. One of the strengths of decision trees is their versatility, they can be used for both classification and regression tasks, depending on the problem at hand [39]. In regression applications, a decision tree is essentially a structured collection of splits based on threshold values learned from the training dataset. Once the tree is trained, it is applied to validation and test sets to generate predictions, making it a powerful tool for capturing relationships between input parameters and target properties in experimental and simulation-based studies.

#### 2.4.2. Linear Regression

Linear regression (LR) is one of the most widely used predictive modeling techniques in machine learning and statistical analysis [40]. LR is widely employed across various fields, including engineering, economics, and scientific research, due to its interpretability and computational efficiency. It is a fundamental approach for modeling relationships between a dependent variable and one or more independent variables by fitting a linear equation to the observed data. The primary objective of LR is to minimize the difference between predicted and actual values, typically achieved using the least squares minimization method. Mathematically, the linear regression model is expressed as:

(6) $$Y=\beta_{o}+\sum_{n=1}^{N}\beta_{n}X_{n}+\varepsilon$$

where *Y* is the dependent variable, *X* is the independent variable, $\beta_{o}$ is the intercept (value of *Y* when *X* = 0), $\beta_{n}$ is the vector of coefficients, and ε is the error term that captures randomness and unobserved factors.

#### 2.4.3. Ridge Regression

Ridge regression (RR) is an extension of linear regression that incorporates L_2_ regularization to address multicollinearity and overfitting issues [41]. Unlike standard linear regression, which minimizes the residual sum of squares (RSS) to estimate regression coefficients, ridge regression adds a penalty term proportional to the L_2_ norm of the coefficient vector. This additional constraint discourages excessively large coefficients, improving the model’s generalization ability, especially when dealing with highly correlated predictors. The ridge regression estimator is obtained by minimizing the following loss function:

(7) $${\hat{\beta }}^{ridge}=arg{min}_{\beta }{\left\|Y-X\beta \right\|}_{2}^{2}+\lambda {\left\|\beta \right\|}_{2}^{2}$$

(8) $${\left\|Y-X\beta \right\|}_{2}^{2}=\Sigma_{i=1}^{n}{\left({Y_{i}X}_{i}^{T}\beta \right)}^{2}$$

$\hat{\beta }$ is the estimated regression coefficients, β is the vector of regression coefficients to be estimated, ${\left\|\beta \right\|}_{2}^{2}$ is the L_2_ norm, ${\left\|Y-X\beta \right\|}_{2}^{2}$ is the residual sum of squares (RSS), measures the difference between predicted and observed values, and λ is the regularization parameter that controls the strength of the penalty. Higher values of λ lead to more coefficients to zero.

#### 2.4.4. Lasso Regression

Lasso Regression (LAR) is another extension of linear regression that incorporates L_1_ regularization, which introduces sparsity into the model by selectively reducing some regression coefficients to zero [42]. This makes Lasso particularly useful for feature selection in high-dimensional datasets, as it can effectively remove less important predictors, leading to a more interpretable model. The Lasso regression estimator is obtained by minimizing the following objective function:

(9) $${\hat{\beta }}^{lasso}=arg{min}_{\beta }{\left\|Y-X\beta \right\|}_{2}^{2}+\lambda {\left\|\beta \right\|}_{1}$$

(10) $${\left\|\beta \right\|}_{1}=\sum_{j=1}^{p}\left|\beta_{j}\right|$$

where ${\left\|\beta \right\|}_{1}$ is the L_1_ norm of the coefficient vector, which penalizes the absolute size of coefficients.

#### 2.4.5. Elastic Nets

Elastic Net (EN) is a sophisticated extension of both Ridge regression and Lasso regression. It combines the strengths of both methods by integrating L_1_ regularization (from Lasso) and L_2_ regularization (from Ridge) into a single model [43]. This hybrid approach allows Elastic Net to adapt to various types of data and better handle situations where predictors are highly correlated or when the number of predictors exceeds the number of observations. By balancing the effects of Lasso and Ridge, Elastic Net benefits from the sparsity of Lasso and the stability of Ridge. In contrast to Lasso, which may struggle with highly correlated predictors by arbitrarily selecting one and discarding others, or Ridge, which cannot set coefficients to zero, Elastic Net provides a robust solution for feature selection and coefficient shrinkage. This ability makes it especially useful in scenarios where there are multiple correlated features, which is common in high-dimensional datasets such as genomics or finance. The mathematical formulation of Elastic Net regression is given by the following loss function:

(11) $${\hat{\beta }}^{EN}=\left(1+\frac{\lambda_{2}}{n}\right)\left\{arg{min}_{\beta }{\left\|Y-X\beta \right\|}_{2}^{2}+\lambda_{2}{\left\|\beta \right\|}_{2}^{2}+\lambda_{1}{\left\|\beta \right\|}_{1}\right\}$$

where ${\hat{\beta }}^{EN}$ represents the estimated regression coefficients, *Y* is the vector of observed dependent variable values, *X* is the matrix of independent (predictor) variables, ${\left\|\beta \right\|}_{1}$ is the L_1_ norm, which is the sum of the absolute values of the coefficients $\sum_{j=1}^{P}\left|\beta_{j}\right|$, encouraging sparsity and feature selection,${\left\|\beta \right\|}_{2}^{2}$ is the L_2_ norm, which is the sum of the squares of the coefficients, promoting coefficient shrinkage and reducing multicollinearity, $\lambda_{1}\;$ is the L_1_ regularization parameter, which controls the strength of the penalty for sparsity, $\lambda_{2}$ is the L_2_ regularization parameter, controlling the strength of the penalty for coefficient shrinkage, *n* is the number of observations in the dataset.

#### 2.4.6. Multilayer Perceptron

Multilayer Perceptron (MLP), also called a feedforward neural network, is a form of ANN consisting of multiple layers of neurons interconnected with each other, including input, hidden, and output layers, where each layer contains a set of perception elements known as neurons. The input data is processed through the layers only in one direction, moving from the input to the output layer without loops [44]. Each neuron processes its inputs using an activation function, such as Rectified Linear Unit (ReLU), which helps the network learn complex patterns. ReLU is widely used because it introduces non-linearity while keeping computations efficient. It outputs the input directly if it is positive, but returns zero for negative inputs. This allows the network to map data more effectively by preserving important features and enabling deeper networks to learn without the vanishing gradient problem. The combination of weighted connections, biases, and ReLU activation ensures the network can adapt and refine its mapping process during training.

**Figure 3:**
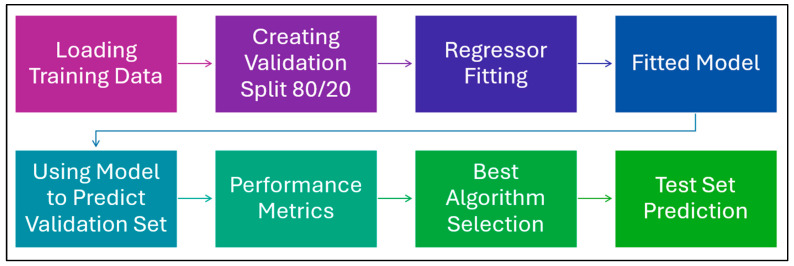
ML Pipeline to implement the algorithms.

To sum up, Figure 3 below describes the pipeline followed to implement the ML algorithms that are described above. In addition, Table 3 details the hyperparameters (HP) for each of these algorithms as implemented in the pipeline.

## 3. Results and Discussion

### 3.1. Experimental Results

From the quasi-static compression test, key crashworthiness indicators were evaluated, including initial peak load, crushing mean load, and energy absorption. These indicators provide critical insights into the structural performance and energy dissipation capabilities of the tested specimens under compressive loading. The calculated values for these indicators are summarized in Table 4. Following the experimental phase, the obtained crashworthiness data was utilized to train and validate ML models. The ML models were developed to analyze and predict the crashworthiness behavior of similar structures, leveraging the experimental dataset to enhance their accuracy and generalization capability. This data-driven approach enables the models to identify patterns and relationships between structural parameters and crash performance, potentially aiding in the design and optimization of energy-absorbing structures.

**Figure 4:**
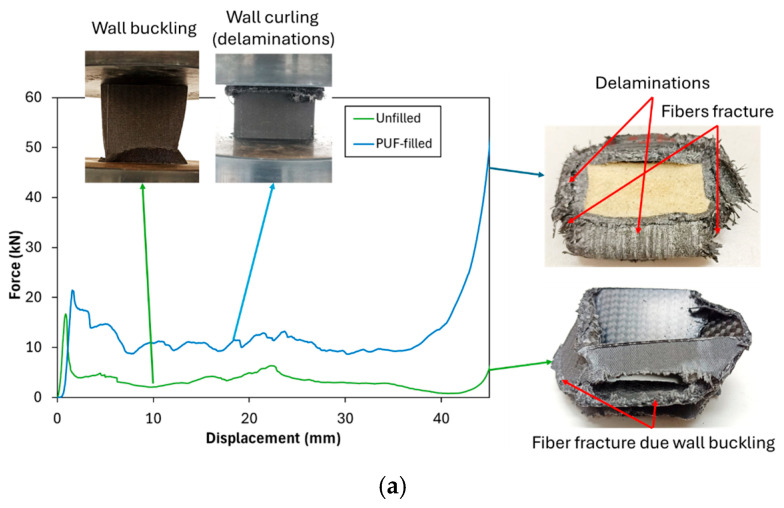

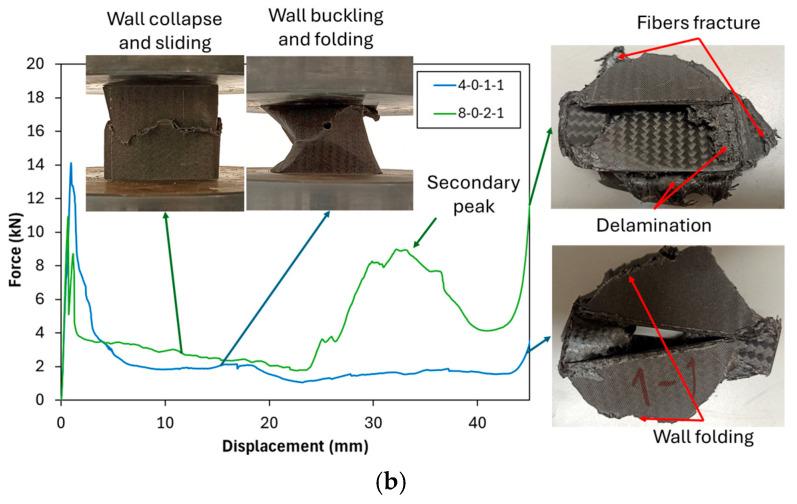
Crushing test of (**a**) comparison of unfilled and PUF-filled neat tubes and (**b**) Perforated tube (4-0-1-1 and 8-0-2-1).

The experimental results declared that the addition of PUF to neat (unperforated) tubes resulted in a significant improvement in crashworthiness indicators compared to their unfilled counterparts. The presence of PUF contributed to an enhanced *EA* capacity and improved deformation control, both of which are crucial for increasing structural resistance under loading conditions. The addition of PUF to the inner part of the CFRP tube has ability to support the wall and prevent the localized buckling in the wall of the tube. The *P_m_* and *EA* of neat PUF-filled tubes exhibited an approximately three-fold increase relative to the unfilled tubes, demonstrating the effectiveness of PUF in reinforcing structural integrity. Furthermore, the failure mode of the tubes underwent a notable transformation. In unfilled tubes, failure was primarily characterized by localized wall buckling. Meanwhile, in PUF-filled tubes, the failure mechanisms evolved to include curling, delamination, and fiber fracture [45], as depicted in Figure 4a. These latter-mentioned failure modes generally require higher force levels, leading to a significant enhancement in *EA*. The increased resistance to deformation in PUF-filled tubes underscores the beneficial role of PUF as a reinforcement material in CFRP that fails by localized buckling, making it a viable solution for improving crashworthiness in structural applications.

Meanwhile, the introduction of holes in unfilled tubes influenced all crashworthiness indicators differently depending on the hole size. Specifically, at a 4 mm hole diameter (4-0-1-1), a reduction in all crashworthiness indicators was observed. The reduction occurs because adding a 4 mm hole does not alter the failure mode but instead reduces the buckling force, as explained by the decrease in Pm compared to the neat unfilled tube (0-0-0-0), as can be seen in Table 4 and Figure 4b. However, at 6 mm and 8 mm hole diameters, the crashworthiness indicators exhibited an increasing trend, suggesting a complex interaction between structural integrity and localized stress distribution. As shown in Figure 4b, the 8-0-2-1 configuration demonstrated that failure mode alteration occurs. The failure was initiated with a wall collapse due to micro-buckling at the hole site, causing the cross-section to separate into upper and lower parts; however, it remained aligned without wall bending. This initial collapse was followed by wall sliding between the upper and lower sections of the tube. Once the collapsed portion of the tube reached the compression plate, the load increased again, producing a secondary peak. Fiber fracture and slight delamination appeared as a result of the wall sliding process. These behaviors contributed to an improvement in both the mean crushing force (Pm) and the energy absorption (EA).

For PUF-filled tubes, the presence of holes led to a decrease in crashworthiness indicators across all specimens when compared to neat (unperforated) PUF-filled tubes. This reduction indicates that the introduction of perforations in PUF-filled structures compromises their ability to effectively absorb energy and maintain structural stability under compression.

**Figure 5:**
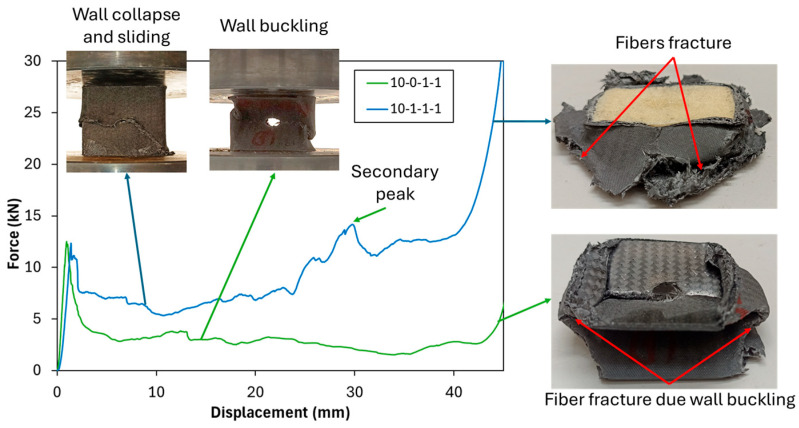
Crushing test comparison of unfilled and PUF-filled with 10 mm hole tubes.

A direct comparison between different tube configurations highlights the positive impact of PUF filling on crashworthiness indicators, particularly in hole configurations 10-0-1-1 and 10-1-1-1. In these configurations, the inclusion of PUF led to notable improvements in *P_m_* and *EA*. The observed deformation mechanism reveals that, in the 10-0-1-1 configuration (unfilled tube), wall buckling similar to that of the neat unfilled tube was observed. Meanwhile, in 10-1-1-1 configuration (PUF-filled tubes), micro-buckling is the initial mode of wall collapse on the hole area [46], followed by wall sliding, as illustrated in Figure 5. Furthermore, the configuration exhibited a secondary peak load, attributed to wall sliding that then contacted the compression surface, leading to an increase in load and consequently enhancing both the *P_m_* and *EA*. These suggest that PUF filling plays an essential role in stabilizing the deformation process by mitigating buckling and bending, thereby enhancing the uniformity of energy dissipation during compression. The presence of PUF effectively redistributes the crushing forces throughout the tube structure, stabilizing the walls and reducing wall buckling, which in turn enhances crashworthiness performance. By reinforcing the tube’s structural integrity, PUF filling ensures a more controlled and progressive energy dissipation mechanism, making it an effective strategy for improving the impact resistance of lightweight structural components.

**Figure 6:**
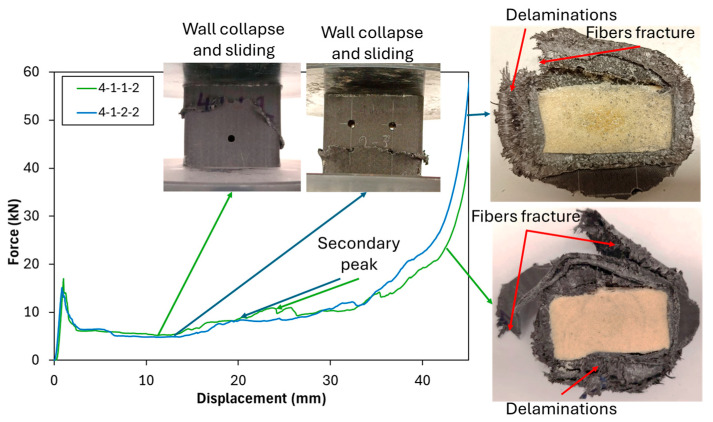
Crushing test comparison of PUF-filled with the addition of a 4 mm hole in the x-direction.

Figure 6 shows the comparison of the crushing characteristics profile with the addition of a 4 mm hole in the x-direction. Adding a 4 mm hole diameter in the x-direction, as seen in specimens 4-1-1-2 and 4-1-2-2, reduces the *P_ip_*. A lower *P_ip_* indicates a decrease in the maximum force that can be handled by the structure during crushing. However, a reduction in *P_ip_* must be carefully evaluated alongside other crashworthiness parameters. In this case, the reduction in *P_ip_*, combined with a similar *P_m_*, contributes to an improvement in *CFE*, meaning that the structure is able to sustain a more stable load during crushing. This is a desirable characteristic in crashworthiness design, as it ensures energy is absorbed more efficiently rather than being transferred to other structures. Furthermore, *EA* values also show consistent results. The introduction of holes in the x-direction did not significantly alter the energy dissipation capacity of the structure. An increase in the number of holes in the x-direction reduces the *P_ip_* due to the reduction in the cross-section of the tube, on the area of the hole, reducing the ability of the tube to handle the compression load. However, after crushing the hole part enclosed, the tube will behave similarly. Failure mode showing similar behavior, started from micro-buckling, which led to wall collapse, followed by sliding of the wall, which then increased the load (secondary peak).

**Figure 7:**
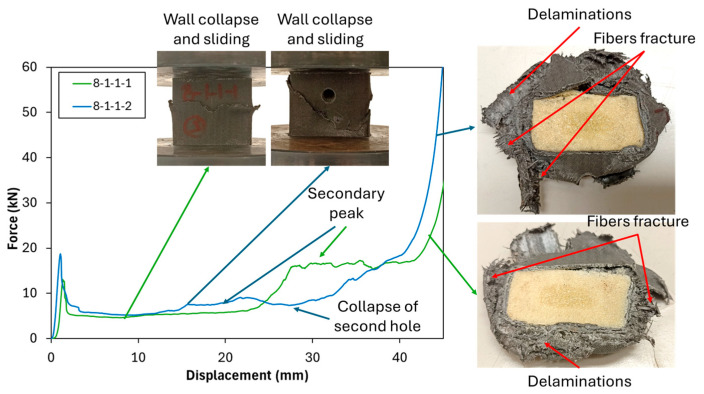
Crushing test comparison of different hole numbers on y-direction for PUF-filled tubes at 8 mm hole.

Additionally, Figure 7 demonstrates the comparison of tubes with different hole numbers in the y-direction of 8 mm holes. The addition of holes in the y-direction, as observed in specimens 8-1-1-1 and 8-1-1-2, results in an increase in *P_ip_* while reducing both *P_m_* and *EA*. Both show an increase in *P_ip_*, suggesting that the tube experiences a higher initial peak force, followed by a sudden drop. The reduction in *P_m_* and *EA* further indicates that these hole configurations in the y-direction negatively affect the energy absorption capacity of the tube at an 8 mm hole diameter configuration. This may be due to the weakening of the structural integrity in the vertical direction, making the tube less efficient at distributing crashing energy across its wall. The presence of the second hole in the y-direction reduces the energy absorbed from the secondary peak due to the secondary collapse from the hole.

An increase in hole diameter, as observed in specimens 8-1-1-1 (Figure 7) and 10-1-1-1 (Figure 5), shows a decrease in *P_ip_* while the *EA* and *P_m_* values remain nearly similar. A higher hole diameter tends to reduce the *P_ip_* due to the reduction in the cross-section of the tube wall on the area of the hole, reducing the ability of the tube to handle the compression load, which is a similar behavior observed with the addition of a hole in the x-direction.

Initial failure type influences the overall crashworthiness performance. Buckling failure has less ability to dissipate energy effectively, influencing the deformation mode and overall crashworthiness performance [47,48]. The introduction of holes alters the stress distribution, affecting whether the failure mode is more dominated by buckling, wall folding, wall collapse, curling, or delamination. A combination of different failures effectively absorbed more energy compared to pure wall buckling failure. The interaction between PUF and hole placement plays a fundamental role in determining the structural behavior under crushing. Optimizing the balance between hole placement, reinforcement materials, and tube geometry is essential in designing structures with enhanced energy absorption capabilities while maintaining adequate structural integrity.

### 3.2. Machine Learning Prediction

Table 5 shows the validation RMSE and MAPE results for each of the utilized ML algorithms across the 10 different samples, as mentioned in the methodology section. From the average RMSE and MAPE values, it is evident that the DTR emerged as the best-performing algorithm, achieving the lowest MAPE of 11.37%, indicating a high level of predictive accuracy on the validation dataset. Conversely, the MLP exhibited the highest MAPE of 93.5%, making it the least effective model among the tested algorithms. The poor performance of MLP suggests that it struggled to capture the underlying patterns in the dataset, possibly due to overfitting, suboptimal hyperparameters, or the need for more training data.

**Figure 8:**
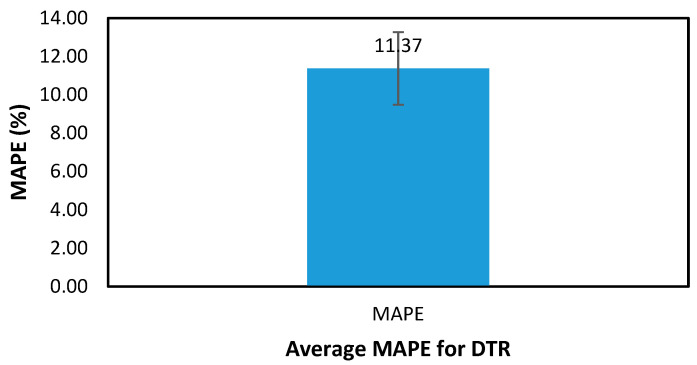
Validation comparison on sample 1.

Confidence interval across samples of the best performing algorithm is shown in Figure 8 for the MAPE results, where the average is 11.37 ± 1.89. This creates a confidence interval for MAPE values of DTR in the range (9.48, 13.26), which demonstrates the robustness of the results.

It is clear that MLP was the worst-performing algorithm on the validation sets, both in individual samples and on the overall average. This is due to MLP’s model complexity, given the small size of the dataset. It is well established that MLP requires huge datasets to converge to the optimal solution in its iterative weight update of the neural network. Nonetheless, MLP was used in this paper to demonstrate this fact of its weakness when applied to small datasets for comparison purposes.

As mentioned, DTR was the best-performing algorithm on the validation data. Given the size of the data and the minimal model complexity of the DTR algorithm, overfitting may not be considered an issue. The model hyperparameters are kept at defaults for all algorithms due to the purpose of comparison. Future research may include hyperparameter optimization for the best-performing algorithm.

As seen in Table 5, DTR is the best-performing algorithm. Prediction vs. truth values for the best performing algorithm, DTR, on the validation sample 1, where MAPE = 6.9% is shown in Table 6.

**Figure 9:**
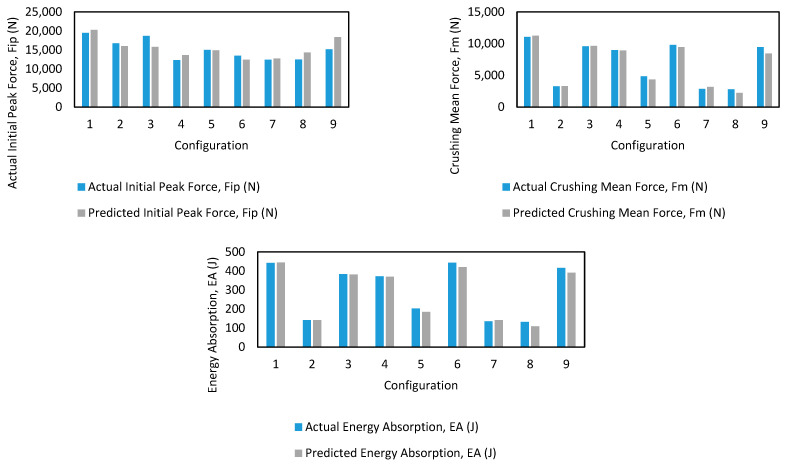
Validation comparison on sample 1 for each output.

In addition, Figure 9 below demonstrates the comparisons between actual and predicted values on the validation sample 1 for each of the three outputs. The differences between actual and predicted values seem minimal for this sample. It is important to note that this is only one sample and used here for demonstration purposes; however, it still provides insight into the potential of ML approaches to alleviate the need for costly experiments.

**Figure 10:**
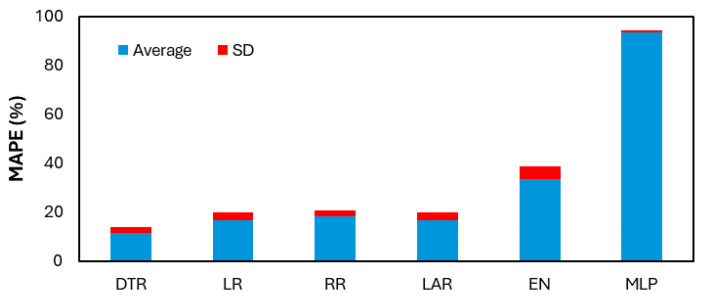
Validation MAPE comparison of ML algorithms.

As DTR is the best-performing ML algorithm, it was used to predict the output of the testing set, as shown in Table 7 for 20 input combinations. DTR was used to predict 10 different samples of combination splits (training and validation) in this testing stage. The predictions shown in Table 7 are the averages of these 10 predictions per input combination. The prediction from DTR shows reasonable results compared to the experimental results. It is worth mentioning that Table 7 provides a unique perspective on the capability of machine learning algorithms in the area of crash box testing. ML reduces the reliance on and necessity to perform costly and time-consuming experimentation by seamlessly predicting reasonable initial peak force, crushing mean force, and energy absorption based on intermediate configurations. Figure 10 below compares the average and standard deviation across all six utilized algorithms in the validation stage, showcasing DTR as the best-performing algorithm.

## 4. Conclusions

This paper investigates the effect of holes on the crashworthiness performance of rectangular tubes made of CFRP, both with and without PUF-filled inner structures. The designed tubes were subjected to quasi-static axial compression loading. ML was employed to predict the crashworthiness properties of the crash boxes in the presence of holes and PUF filling. From the experimental work, the findings in this paper emphasized the intricate relationship between PUF addition, hole placement, and crashworthiness indicators. PUF significantly enhanced crashworthiness by stabilizing deformation and increasing *EA* and *CFE* by 213% and 175%, respectively. The presence of holes can either improve or weaken specific performance indicators. Hole diameters and arrays were also found to influence performance strongly. In unfilled tubes, small holes of 4 mm reduced all crashworthiness indicators. In comparison, holes of 6–8 mm tended to lead to increases in *EA* and *CFE*, with maximum increases of 130% and 76%, respectively, for the 8-0-2-1 configuration. In contrast, for PUF-filled tubes, the introduction of holes constantly reduced the crashworthiness compared to neat PUF-filled tubes. Furthermore, the failure mechanisms of the tubes were notably affected by the presence of PUF. Neat unfilled tubes failed primarily through wall buckling, and PUF-filled tubes without holes demonstrated more progressive failure modes such as curling, delamination, and fiber fracture. Meanwhile, the addition of holes has altered the failure mechanism into micro-buckling and wall sliding. Understanding these effects is essential for designing energy-absorbing structures with optimized crashworthiness for automotive, aerospace, and other protective structural components applications.

From the ML analysis, the DTR method showed the lowest values of RMSE and MAPE for 1251 and 11.37%, respectively, and was selected as the best ML method to predict the crashworthiness indicators of the tubes in the configurations that were not experimentally performed (test set). The size of the data that was used for the training and validation may reduce the validity of the prediction results on the test set. However, the practical engineering significance of the ML prediction framework provides a substantial contribution to reducing the need for further costly experimentation. The ML test results provide insight into the crashworthiness without the need for repeating the experiment with different parameters.

Future research can expand on this work in several directions. The effect of different geometries, such as height, cross-sectional dimensions, shapes, and wall thickness, could be further optimized. The impact of environmental factors such as temperature and moisture, etc., on the crashworthiness could be further explored. Combining the finite element method (FEM) and ML could be another approach for predicting and optimizing the crashworthiness of CFRP tubes.

## Figures and Tables

**Table 1:** Experimental setup specimen configurations.

No.	Configuration	Hole Diameter (mm)	PUF-Filled	Number of Holes on x-Direction	Number of Holes on y-Direction
1	0-0-0-0	0	0	0	0
2	0-1-0-0	0	1	0	0
3	4-0-1-1	4	0	1	1
4	4-1-1-1	4	1	1	2
5	4-1-2-2	4	1	2	2
6	6-0-1-2	6	0	1	2
7	6-0-2-2	6	0	2	2
8	6-1-2-1	6	1	2	1
9	8-0-2-1	8	0	2	1
10	8-1-1-1	8	1	1	1
11	8-1-1-2	8	1	1	2
12	10-0-1-1	10	0	1	1
13	10-0-2-2	10	0	2	2
14	10-1-1-1	10	1	1	1

**Table 2:** Testing dataset.

Configuration	Hole Diameter (mm)	PUF-Filled	Number of Holes on x-Direction	Number of Holes on y-Direction
4-0-1-2	4	0	**1**	2
4-0-2-1	4	0	**2**	1
4-0-2-2	4	0	2	2
6-0-1-1	6	0	1	1
6-0-2-1	6	0	2	1
8-0-1-1	8	0	1	1
8-0-1-2	8	0	1	2
8-0-2-2	8	0	2	2
10-0-2-1	10	0	2	1
10-0-1-2	10	0	1	2
4-1-2-1	4	1	2	1
4-1-1-1	4	1	1	1
6-1-1-1	6	1	1	1
6-1-1-2	6	1	1	2
6-1-2-2	6	1	2	2
8-1-2-1	8	1	2	1
8-1-2-2	8	1	2	2
10-1-2-2	10	1	2	2
10-1-2-1	10	1	2	1
10-1-1-2	10	1	1	2

**Table 3:** Hyperparameter selection for each of the six ML algorithms.

DTR		LR		RR		LAR		EN		MLP	
HP	Value	HP	Value	HP	Value	HP	Value	HP	Value	HP	Value
Criterion	Squared error	Fit intercept	True	Fit intercept	True	Fit intercept	True	Fit intercept	True	Hidden layers	1
Splitter	Best	Tol	0.000001	Tol	0.0001	Tol	0.0001	Tol	0.0001	Tol	0.0001
Min samples split	2	Copy X	True	Copy X	True	Copy X	True	Copy X	True	Neurons	100
Min samples leaf	1	Positive	False	Positive	False	Positive	False	Positive	False	Activation	ReLu
				Solver	Auto	Selection	Cyclic	Selection	Cyclic	Solver	Adam
				Alpha	1	Alpha	1	Alpha	1	beta_1	0.9
								L1 ratio	0.5	beta_2	0.999

**Table 4:** Experimental results.

Configuration *	Hole Diameter (mm)	PUF-Filled	Number of Holes on x-Direction	Number of Holes on y-Direction	Initial Peak Load, *P_ip_* (N)	Crushing Mean Load, *P_m_* (N)	Energy Absorption, EA (J)
0-0-0-0	0	0	0	0	16,700	3313	141
0-0-0-0	0	0	0	0	15,305	3307	142
0-0-0-0	0	0	0	0	16,735	3274	142
0-1-0-0	0	1	0	0	19,490	11,066	442
0-1-0-0	0	1	0	0	21,480	11,080	439
0-1-0-0	0	1	0	0	19,075	11,392	450
4-0-1-1	4	0	1	1	14,120	1926	96
4-0-1-1	4	0	1	1	12,470	2742	119
4-0-1-1	4	0	1	1	14,100	2819	124
4-1-1-2	4	1	1	2	17,875	6848	278
4-1-1-2	4	1	1	2	17,000	9178	366
4-1-1-2	4	1	1	2	15,275	10,126	402
4-1-2-2	4	1	2	2	15,125	9134	363
4-1-2-2	4	1	2	2	16,825	8993	359
4-1-2-2	4	1	2	2	12,915	7116	283
6-0-1-2	6	0	1	2	14,340	3757	162
6-0-1-2	6	0	1	2	15,465	4898	207
6-0-1-2	6	0	1	2	15,025	4853	203
6-0-2-2	6	0	2	2	10,900	3275	144
6-0-2-2	6	0	2	2	12,420	2860	135
6-0-2-2	6	0	2	2	14,560	3071	139
6-1-2-1	6	1	2	1	13,165	9356	367
6-1-2-1	6	1	2	1	18,440	9941	396
6-1-2-1	6	1	2	1	18,670	9565	383
8-0-2-1	8	0	2	1	10,935	4266	199
8-0-2-1	8	0	2	1	11,165	5245	239
8-0-2-1	8	0	2	1	13,450	6780	311
8-1-1-1	8	1	1	1	13,070	9497	416
8-1-1-1	8	1	1	1	11,840	9428	425
8-1-1-1	8	1	1	1	13,505	9800	443
8-1-1-2	8	1	1	2	18,725	8581	398
8-1-1-2	8	1	1	2	15,185	9441	416
8-1-1-2	8	1	1	2	18,065	8310	383
10-0-1-1	10	0	1	1	13,970	2274	109
10-0-1-1	10	0	1	1	14,730	2164	109
10-0-1-1	10	0	1	1	12,515	2788	132
10-0-2-2	10	0	2	2	9440	3167	155
10-0-2-2	10	0	2	2	7285	2794	134
10-0-2-2	10	0	2	2	9430	3838	165
10-1-1-1	10	1	1	1	12,350	8977	372
10-1-1-1	10	1	1	1	12,905	7953	333
10-1-1-1	10	1	1	1	14,440	9853	407

* Notes: for example, the symbol 8-1-1-2 denotes an 8 mm hole diameter, with PUF-filled, one hole in x-direction, and 2 holes in y-direction.

**Table 5:** Validation results of the used ML techniques.

Algorithm	DTR		LR		RR		LAR		EN		MLP	
Sample	RMSE	MAPE	RMSE	MAPE	RMSE	MAPE	RMSE	MAPE	RMSE	MAPE	RMSE	MAPE
0	1269.87	12.49	1591.08	13.15	1653.99	15.66	1591.93	13.19	2266.92	29.44	9616.76	92.48
1	1015.81	6.90	1202.08	12.97	1186.86	14.37	1200.75	12.90	1699.13	30.23	9853.45	93.78
2	1168.14	11.58	1557.40	19.07	1540.39	20.53	1557.17	19.05	1946.74	33.24	9636.33	94.38
3	1293.61	16.39	1539.34	22.69	1493.15	22.24	1539.61	22.60	1825.48	27.86	9793.56	93.46
4	1308.96	10.25	1179.65	14.17	1199.65	16.27	1179.07	14.07	1892.59	36.57	9349.70	93.27
5	1202.65	14.09	1459.19	16.51	1519.12	19.11	1459.66	16.80	2173.42	34.03	9836.02	94.17
6	1245.53	9.86	1306.97	16.55	1361.01	18.53	1307.18	16.44	2043.18	38.04	9770.82	92.64
7	1791.48	10.98	1716.62	19.77	1717.78	19.64	1716.11	19.29	2146.00	36.65	9460.42	92.80
8	978.11	9.13	1447.69	13.73	1460.02	14.88	1447.92	13.60	1970.49	25.89	9513.69	94.34
9	1240.50	12.06	1455.12	17.10	1463.56	19.23	1455.30	17.05	1982.90	42.01	9123.99	93.72
Average	1251.47	11.37	1445.51	16.57	1459.55	18.05	1445.47	16.50	1994.68	33.39	9595.48	93.50
SD	220.30	2.65	171.73	3.21	172.35	2.61	172.02	3.16	170.21	5.05	236.68	0.70

**Table 6:** Experimental results validation.

Actual			Predicted		
Actual Initial Peak Force, Fip (N)	Actual Crushing Mean Force, Fm (N)	Actual Energy Absorption, EA (J)	Predicted Initial Peak Force, *P_ip_* (N)	Predicted Crushing Mean Force, *P_m_* (N)	Predicted Energy Absorption, EA (J)
19,490	11,066	442	20,277.5	11,236	444.5
16,735	3274	142	16,002.5	3310	141.5
18,670	9565	383	15,802.5	9648.5	381.5
12,350	8977	372	13,672.5	8903	370
15,025	4853	203	14,902.5	4327.5	184.5
13,505	9800	443	12,455	9462.5	420.5
12,420	2860	135	12,730	3173	141.5
12,515	2788	132	14,350	2219	109
15,185	9441	416	18,395	8445.5	390.5

**Table 7:** Prediction results of the DTR.

Configuration	Hole Diameter (mm)	PUF-Filled	Number of Holes in the x Direction	Number of Holes in the y Direction	Initial Peak Force, *P_ip_* (N)	Crushing Mean Force, *P_m_* (N)	Energy Absorption, EA (J)
4-0-1-2	4	0	1	2	14,903	4339	184
4-0-2-1	4	0	2	1	12,387	3378	154
4-0-2-2	4	0	2	2	12,820	3069	139
6-0-1-1	6	0	1	1	13,755	2729	122
6-0-2-1	6	0	2	1	12,387	3378	154
8-0-1-1	8	0	1	1	13,870	2611	124
8-0-1-2	8	0	1	2	14,313	4344	186
8-0-2-2	8	0	2	2	12,034	4828	222
10-0-2-1	10	0	2	1	9209	3626	165
10-0-1-2	10	0	1	2	14,192	4135	178
4-1-2-1	4	1	2	1	16,127	9352	371
4-1-1-1	4	1	1	1	15,623	9354	385
6-1-1-1	6	1	1	1	15,636	9625	395
6-1-1-2	6	1	1	2	16,729	8988	359
6-1-2-2	6	1	2	2	15,394	8711	347
8-1-2-1	8	1	2	1	13,917	9607	416
8-1-2-2	8	1	2	2	15,283	8547	359
10-1-2-2	10	1	2	2	14,625	8542	356
10-1-2-1	10	1	2	1	13,452	9004	372
10-1-1-2	10	1	1	2	15,675	8752	384

## Data Availability

The original contributions presented in this study are included in the article material. Further inquiries can be directed to the corresponding author.

## Abbreviations

The following abbreviations are used in this manuscript:

ANN	Artificial Neural Network
CFE	Crush force efficiency
CFRP	Carbon fiber-reinforced polymer
DTR	Decision tree regressor
EA	Energy absorption
EN	Elastic nets
HP	Hyperparameters
LAR	Lasso regressor
LR	Linear regressor
MAPE	Mean absolute percentage error
ML	Machine learning
MLP	Multi-layer perceptron
NSGA-II	Non-dominated sorting genetic algorithm II
Pip	Initial peak force
Pm	Mean crushing force
PUF	Polyurethane foam
ReLU	Rectified Linear Unit
RL	Reinforcement learning
RMSE	Root mean squared error
RNN	Recurrent Neural Networks
RR	Ridge regressor
RSS	Residual sum of squares
SEA	Specific absorbed energy
Vf	Fiber volume fraction

## References

[1] Baroutaji A., Morris E., Olabi A.G. (2014). Quasi-Static Response and Multi-Objective Crashworthiness Optimization of Oblong Tube under Lateral Loading. Thin-Walled Struct..

[2] Awd Allah M.M., Abd El-baky M.A. (2024). Multi-Objective Optimization through Desirability Function Analysis on the Crashworthiness Performance of Thermoplastic/Thermoset Hybrid Structures. Compos. B Eng..

[3] Capretti M., Del Bianco G., Giammaria V., Boria S. (2024). Natural Fibre and Hybrid Composite Thin-Walled Structures for Automotive Crashworthiness: A Review. Materials.

[4] Falaschetti M.P., Semprucci F., Birnie Hernández J., Troiani E. (2025). Experimental and Numerical Assessment of Crashworthiness Properties of Composite Materials: A Review. Aerospace.

[5] Ramalingam S.K., Selvan D.K., Manoj Mohan Prasath V., Suresh Balaji R., Loganathan P., Naveen Kumar A. (2026). A Crashworthiness Study of Composites for Automobiles. Sustainable Composites for Automotive Engineering.

[6] El-baky M.A.A., Allah M.M.A., Kamel M., Abd-Elaziem W. (2022). Lightweight Cost-Effective Hybrid Materials for Energy Absorption Applications. Sci. Rep..

[7] El-baky M.A.A., Allah M.M.A., Kamel M., Abdel-Aziem W. (2023). Fabrication of Glass/Jute Hybrid Composite over Wrapped Aluminum Cylinders: An Advanced Material for Automotive Applications. Fibers Polym..

[8] Al Antali A., Umer R., Zhou J., Cantwell W.J. (2017). The Energy-Absorbing Properties of Composite Tube-Reinforced Aluminum Honeycomb. Compos. Struct..

[9] Allah M.M.A., Hegazy D.A., Alshahrani H., Sebaey T.A., El-baky M.A.A. (2023). Fiber Metal Laminates Based on Natural/Synthesis Fiber Composite for Vehicles Industry: An Experimental Comparative Study. Fibers Polym..

[10] Guo S., Qi J., Wang Y., Liu Z., Li J. (2025). A Flexible Impact Sensor of Interpenetrating-Phase Composite Architecture with High Mechanical Stability and Energy-Absorbing Capability. Adv. Funct. Mater..

[11] Awd Allah M.M., Shaker A., Hassan M.A., Abd El-baky M.A. (2022). The Influence of Induced Holes on Crashworthy Ability of Glass Reinforced Epoxy Square Tubes. Polym. Compos..

[12] Ali-Eldin S.S., El-Moezz S.M.A., Megahed M., Abdalla W.S. (2019). Study of Hybridization Effect of New Developed Rice Straw Mat/ Glass Fiber Reinforced Polyester Composite. J. Nat. Fibers.

[13] Abd El-Aziz K., Megahed M., Saber D. (2024). Mechanical Properties and Corrosion Protection Performance of Micro/Nano Alumina Fillers Coated Steel. Polym. Compos..

[14] Xu Y., Zhang F., Zhai W., Cheng S., Li J., Wang Y. (2022). Unraveling of Advances in 3D-Printed Polymer-Based Bone Scaffolds. Polymers.

[15] El-Baky M.A.A., Allah M.M.A., Kamel M., Abd-Elaziem W. (2022). Energy Absorption Characteristics of E-Glass/Epoxy over-Wrapped Aluminum Pipes with Induced Holes: An Experimental Research. Sci. Rep..

[16] Somwanshi A., Kaware K., Sulakhe V., Kotambkar M., Vasava A. (2025). Advances in Composite Crashworthiness: Material Properties, Failure Mechanisms, and Design Strategies. Eng. Res. Express.

[17] Ozkan D., Gok M.S., Karaoglanli A.C. (2020). Carbon Fiber Reinforced Polymer (CFRP) Composite Materials, Their Characteristic Properties, Industrial Application Areas and Their Machinability.

[18] El Aal M.I.A., Allah M.M.A., El-baky M.A.A. (2023). Carbon-glass Reinforced Epoxy Hybrid Composites for Crashworthy Structural Applications. Polym. Compos..

[19] Alshahrani H., Almeshari B., El-baky M.A.A., Sebaey T.A. (2024). Crashworthiness Assessment of Foam-Filled Internally Strengthened Carbon Fibre-Reinforced Composite Tubes under Axial Compression. Int. J. Crashworthiness.

[20] Renreng I., Djamaluddin F., Mar’uf M., Li Q. (2024). Optimization of Crashworthiness Design of Foam-Filled Crash Boxes under Oblique Loading for Electric Vehicles. Front. Mech. Eng..

[21] Akbari P., Zamani M., Mostafaei A. (2024). Machine Learning Prediction of Mechanical Properties in Metal Additive Manufacturing. Addit. Manuf..

[22] Mobarak M.H., Mimona M.A., Islam M.A., Hossain N., Zohura F.T., Imtiaz I., Rimon M.I.H. (2023). Scope of Machine Learning in Materials Research—A Review. Appl. Surf. Sci. Adv..

[23] Kazi M.-K., Eljack F., Mahdi E. (2022). Design of Composite Rectangular Tubes for Optimum Crashworthiness Performance via Experimental and ANN Techniques. Compos. Struct..

[24] Borse A., Gulakala R., Stoffel M. (2023). Machine Learning Enhanced Optimisation of Crash Box Design for Crashworthiness Analysis. PAMM.

[25] Liang R., Tang X., Huang J., Bastien C., Zhang C., Tuo W. (2024). A Machine Learning-Based Crashworthiness Optimization for a Novel Pine Cone-Inspired Multi-Cell Tubes under Bending. Heliyon.

[26] Sorour S.S., Saleh C.A., Shazly M. (2024). A Review on Machine Learning Implementation for Predicting and Optimizing the Mechanical Behaviour of Laminated Fiber-Reinforced Polymer Composites. Heliyon.

[27] Arnold S.M., Mital S.K., Hearley B.L. Stiffness and Fatigue Life Estimator for Polymer Composite Laminates Using Machine Learning. Proceedings of the American Society for Composites (ASC) 38th Annual Technical Conference.

[28] Chowdhury S.A., Nelon C., Li S., Myers O., Hall A. (2025). Quantification of the Out-of-Plane Loading Fatigue Response of Bistable CFRP Laminates Using a Machine Learning Approach. Mech. Adv. Mater. Struct..

[29] Osa-uwagboe N., Udu A.G., Ghalati M.K., Silberschmidt V.V., Aremu A., Dong H., Demirci E. (2024). A Machine Learning-Enabled Prediction of Damage Properties for Fiber-Reinforced Polymer Composites under out-of-Plane Loading. Eng. Struct..

[30] Allah M.M.A., El-Halim M.F.A., Aal M.I.A.E., El-baky M.A.A. (2024). Picking Up the Optimum Triggering Combinations of Crashworthy 3D-Printed Sustainable Structures: An Experimental Study in Al-Kharj Governorate, KSA. Fibers Polym..

[31] John K.M., Thirumalai Kumaran S. (2020). Backup Support Technique towards Damage-Free Drilling of Composite Materials: A Review. Int. J. Lightweight Mater. Manuf..

[32] Alshahrani H., Sebaey T.A., Awd Allah M.M., Abd El-baky M.A. (2023). Multi-Response Optimization of Crashworthy Performance of Perforated Thin Walled Tubes. J. Compos. Mater..

[33] Pedregosa F., Varoquaux G., Gramfort A., Michel V., Thirion B., Grisel O., Blondel M., Prettenhofer P., Weiss R., Dubourg V. (2011). Scikit-Learn: Machine Learning in Python. J. Mach. Learn. Res..

[34] Van Rossum G., Drake F.L. (2009). Python 3 Reference Manual: (Python Documentation Manual Part 2).

[35] Harris C.R., Millman K.J., van der Walt S.J., Gommers R., Virtanen P., Cournapeau D., Wieser E., Taylor J., Berg S., Smith N.J. (2020). Array Programming with NumPy. Nature.

[36] Haruna S.I., Ibrahim Y.E., Umar I.K. (2025). Machine Learning Approach for Prediction and Reliability Analysis of Failure Strength of U-Shaped Concrete Samples Joined with UHPC and PUC Composites. J. Compos. Sci..

[37] Shaikh A.A., Raheman M.A., Hrairi M., Baig M. (2024). Improving the Performance of Damage Repair in Thin-Walled Structures with Analytical Data and Machine Learning Algorithms. Frat. Integrità Strutt..

[38] Haruna S.I., Ibrahim Y.E., Ahmed O.S., Farouk A.I.B. (2024). Impact Strength Properties and Failure Mode Classification of Concrete U-Shaped Specimen Retrofitted with Polyurethane Grout Using Machine Learning Algorithms. Infrastructures.

[39] Thomas T., Vijayaraghavan A.P., Emmanuel S. (2019). Machine Learning Approaches in Cyber Security Analytics.

[40] James G., Witten D., Hastie T., Tibshirani R., Taylor J. (2023). Statistical Learning.

[41] Rajan M.P. (2022). An Efficient Ridge Regression Algorithm with Parameter Estimation for Data Analysis in Machine Learning. SN Comput. Sci..

[42] Sivakumar N.K., Palaniyappan S., Bodaghi M., Azeem P.M., Nandhakumar G.S., Basavarajappa S., Pandiaraj S., Hashem M.I. (2024). Predictive Modeling of Compressive Strength for Additively Manufactured PEEK Spinal Fusion Cages Using Machine Learning Techniques. Mater. Today Commun..

[43] Malashin I.P., Tynchenko V.S., Nelyub V.A., Borodulin A.S., Gantimurov A.P. (2023). Estimation and Prediction of the Polymers’ Physical Characteristics Using the Machine Learning Models. Polymers.

[44] Chan K.Y., Abu-Salih B., Qaddoura R., Al-Zoubi A.M., Palade V., Pham D.-S., Ser J.D., Muhammad K. (2023). Deep Neural Networks in the Cloud: Review, Applications, Challenges and Research Directions. Neurocomputing.

[45] Ataabadi P.B., Karagiozova D., Alves M. (2019). Crushing and Energy Absorption Mechanisms of Carbon Fiber-Epoxy Tubes under Axial Impact. Int. J. Impact Eng..

[46] Aboudi J., Gilat R. (2022). The Effect of Local and Random Fiber Waviness on the Microbuckling of Composite Materials. Int. J. Solids Struct..

[47] Mamalis A., Robinson M., Manolakos D., Demosthenous G., Ioannidis M., Carruthers J. (1997). Crashworthy Capability of Composite Material Structures. Compos. Struct..

[48] Awd Allah M.M., El-Halim A., Mahmoud F., Abbas M.A., Almuflih A.S., Saleh D.I., El-baky A., Marwa A. (2025). Discovering the Impact of Printing Parameters on the Crashworthiness Performance of 3D-Printed Cellular Structures. Fibers Polym..

